# The effect of improved rural sanitation on diarrhoea and helminth infection: design of a cluster-randomized trial in Orissa, India

**DOI:** 10.1186/1742-7622-9-7

**Published:** 2012-11-13

**Authors:** Thomas Clasen, Sophie Boisson, Parimita Routray, Oliver Cumming, Marion Jenkins, Jeroen H J Ensink, Melissa Bell, Matthew C Freeman, Soosai Peppin, Wolf-Peter Schmidt

**Affiliations:** 1 Faculty of Infectious and Tropical Diseases, London School of Hygiene & Tropical Medicine, London, UK; 2 Water Aid United Kingdom, London, UK; 3 Department of Civil and Environmental Engineering, University of California, Davis, USA; 4 Department of Global Health, Rollins School of Public Health, Emory University, Atlanta, USA; 5 Xavier Institute of Management, Bhubaneswar, India

## Abstract

**Background:**

Infectious diseases associated with poor sanitation such as diarrhoea, intestinal worms, trachoma and lymphatic filariasis continue to cause a large disease burden in low income settings and contribute substantially to child mortality and morbidity. Obtaining health impact data for rural sanitation campaigns poses a number of methodological challenges. Here we describe the design of a village-level cluster-randomised trial in the state of Orissa, India to evaluate the impact of an ongoing rural sanitation campaign conducted under the umbrella of India’s Total Sanitation Campaign (TSC).We randomised 50 villages to the intervention and 50 villages to control. In the intervention villages the implementing non-governmental organisations conducted community mobilisation and latrine construction with subsidies given to poor families. Control villages receive no intervention. Outcome measures include (1) diarrhoea in children under 5 and in all ages, (2) soil-transmitted helminth infections, (3) anthropometric measures, (4) water quality, (5) number of insect vectors (flies, mosquitoes), (6) exposure to faecal pathogens in the environment. In addition we are conducting process documentation (latrine construction and use, intervention reach), cost and cost-effectiveness analyses, spatial analyses and qualitative research on gender and water use for sanitation.

**Results:**

Randomisation resulted in an acceptable balance between trial arms. The sample size requirements appear to be met for the main study outcomes. Delays in intervention roll-out caused logistical problems especially for the planning of health outcome follow-up surveys. Latrine coverage at the end of the construction period (55%) remained below the target of 70%, a result that may, however, be in line with many other TSC intervention areas in India.

**Conclusion:**

We discuss a number of methodological problems encountered thus far in this study that may be typical for sanitation trials. Nevertheless, it is expected that the trial procedures will allow measuring the effectiveness of a typical rural sanitation campaign, with sufficient accuracy and validity.

## Introduction

Diseases associated with poor sanitation cause a large burden of disease worldwide. Diarrhoea alone causes an estimated 4 billion cases and 1.9 million deaths each year among children under 5 years, or 19% of all under-5 deaths in low income settings [1]. Other major diseases associated with poor sanitation are soil-transmitted worm infections, trachoma, lymphatic filariasis and schistosomiasis [2]. In contrast to other Millennium Development Goals, sanitation coverage remains low with 2.5 billion people still lacking access to sanitation. Only 6% of rural residents in India have access to improved sanitation, and about 69% practice open defecation [3].

Systematic reviews have suggested that improved sanitation may reduce diarrhoeal diseases by 22% to 36% [2,4-8]. The studies included in these reviews were observational or small-scale before/after intervention studies that combined sanitation with water supplies or hygiene. The methodological quality of the studies was generally poor [2,5-8]. To date, there is no randomized controlled trial of sanitation interventions to prevent diarrhoeal diseases [2,4-8]. Large RCTs may have been deemed difficult due to logistical constraints, including the long time frame of sanitation campaigns both in terms of construction and the time it takes for behavioural change leading to actual use. Sanitation campaigns are usually conducted by governmental or non-governmental actors. Researchers may have little control over how an intervention is rolled-out once it has started.

Further, the need for sanitation in dense urban areas (ideally by sewage connections) may be uncontroversial, and can be justified on the basis of non-health benefits alone. An RCT may not greatly influence urban sanitation policy. This may be different in rural settings where the health and social benefits are not always obvious to users and where demand for sanitation is often low [9-11]. The fraction of diarrhoea preventable by sanitation may be lower in rural compared to dense urban areas. Current large-scale rural sanitation programmes are conducted in the absence of evidence on its health impact.

In this article we describe the design of a large cluster-randomised trial (CRT) in Orissa (Odisha), India, that seeks to provide evidence on the health impact of sanitation (improved human excreta disposal) in rural low-income settings. We discuss a number of methodological challenges of the trial and our attempts to address these.

## Methods

### Study setting

**Figure 1:**
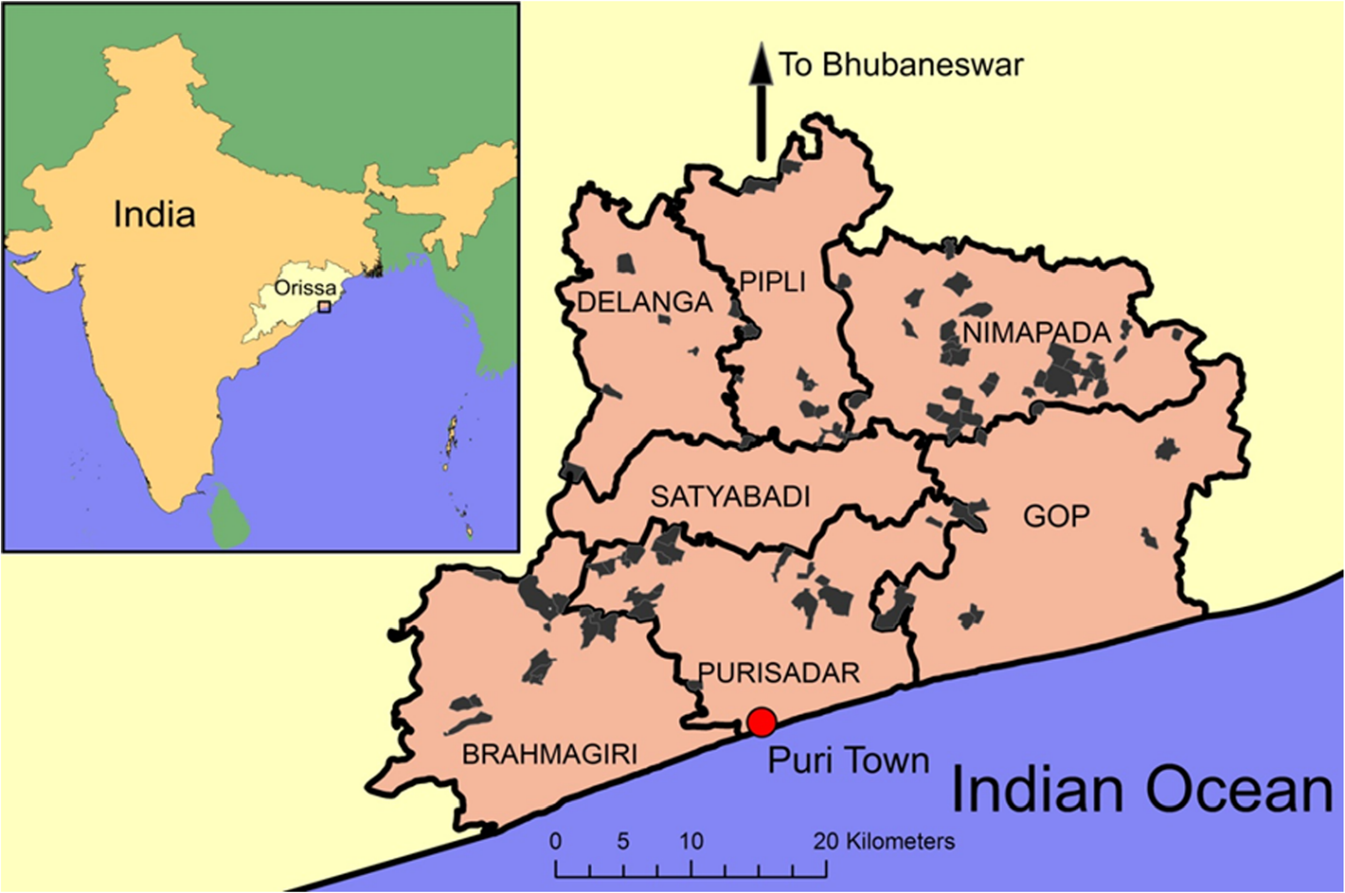
The study area in seven blocks with administrative boundaries of trial villages (grey).

The study is located in Puri, a coastal district in the eastern State of Orissa (Figure 1). More than 50% of the population are recognized by the Indian Government as “below poverty line” (BPL). The area has a tropical climate with a monsoon season from July to September (1500 mm annual rainfall). Puri District is divided into smaller administrative units (Blocks), the unit at which sanitation implementers operate. Agriculture (rice, pulses, vegetables, livestock) is the main source of income. In Puri District, sanitation coverage in 2008 was estimated at 15% in rural areas [12]. In the years preceding the trial, several blocks in Puri had received latrines under the Total Sanitation Campaign (TSC), a long term commitment by the Indian Government to increase sanitation in rural areas [13]. The study is led by researchers at London School of Hygiene and Tropical Medicine and XIMB, with no direct influence on the type and delivery of the intervention.

### Study design

The study has been reviewed by the Ethics Committee of LSHTM, the Xavier Institute of Management, Bhubaneswar and the Asian Institute of Public Health. This trial is registered with ClinicalTrials.gov (Registration No. NCT01214785). The study is a cluster randomized trial with villages as the unit of randomization. Randomisation in clusters was chosen because of the expected community effect of sanitation on disease transmission possibly protecting households without sanitation (“herd immunity”).

**Figure 2:**
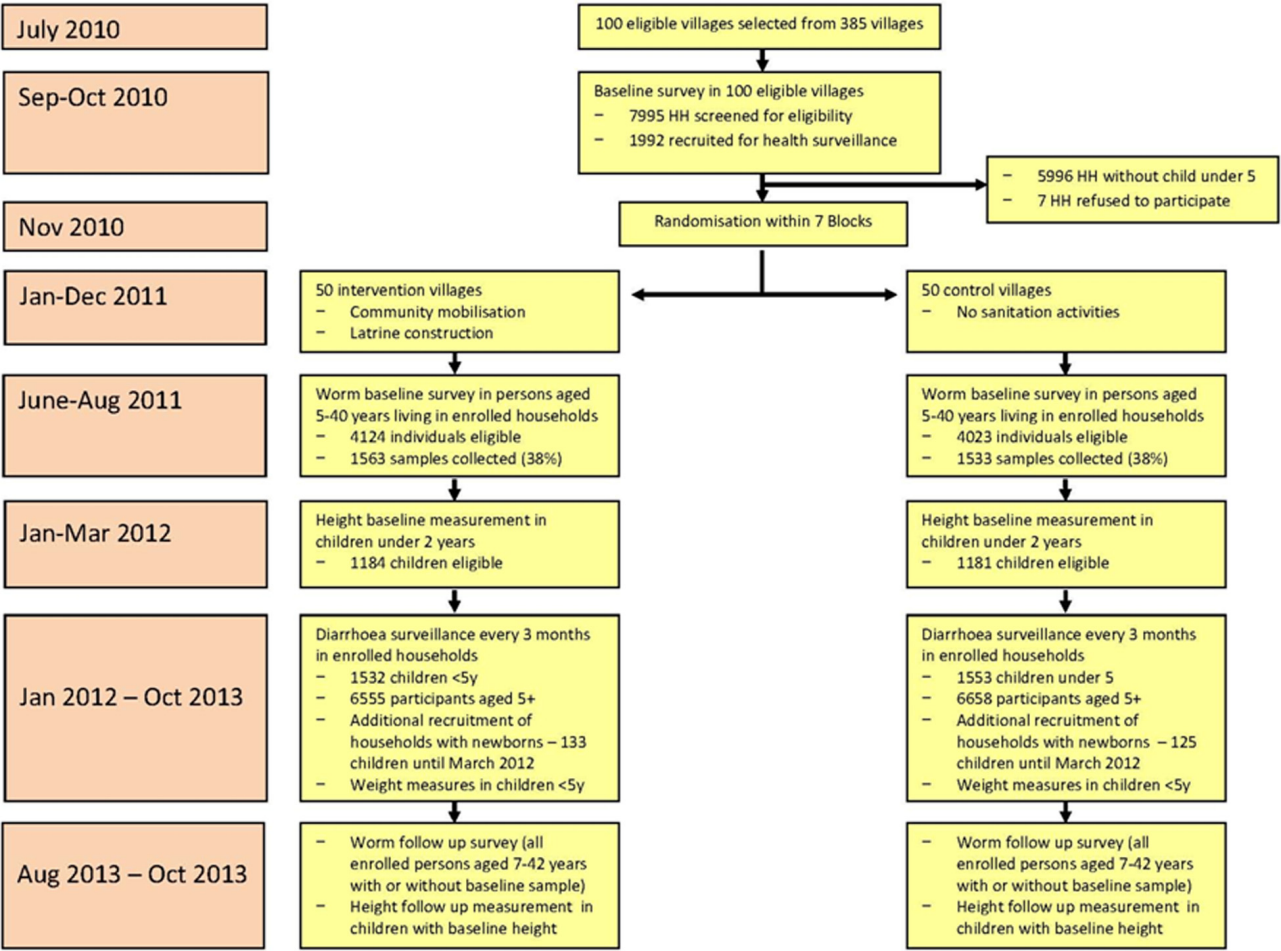
Flow diagram of the study.

From a government list of 385 villages not yet covered by TSC, we selected the first 100 that met the selection criteria (detailed below). We conducted a baseline survey in these villages (Table). Because of the nearly 12-month time for intervention implementation between baseline survey and start of the health outcome surveys, the enrolment procedures had to be repeated in previously enrolled and about 400 of new households (Figure 2).

Following the baseline survey, 50 villages each were randomly allocated to intervention and control in a parallel trial design (Figure 2). The control arm will receive the intervention after trial completion. We also considered a step-wedge design where the intervention roll-out is staggered throughout the follow-up period [14,15]. Step-wedge designs (where only the time point of receiving the intervention is randomised) can be more acceptable to governments and the population than a parallel arm trial. They may also be more robust against unexpected delays in intervention roll-out because follow-up disease surveillance can be started as soon as the first villages have received the intervention. We decided against the step-wedge design because (1) the results of a parallel trial are easier to interpret for policy makers, (2) step-wedge trials require a larger sample size (about 30% or more) [15], and (3) because the NGOs implementing the intervention judged implementation in a parallel trial as feasible.

We specified diarrhoea in children under 5 years as the primary outcome of the study. From the epidemiological point of view, there may be little reason to assume that the effect (relative risk) of sanitation differs by age, since sanitation should lower the overall transmission of pathogens in a community. Including members of all ages of enrolled households in the primary endpoint would have lowered the required sample size. However, the actual sample size increase (number of villages) due to focussing on children under 5 was estimated to be modest (between 10-20%) because diarrhoea is much more common in children and because the gain in study power by increasing the number of individuals *per cluster* is limited [14]. We decided that having sufficient power to detect an effect in children under 5 would contribute to public health policy decision-making and advocacy.

### Sample size calculation

Sample size calculations for CRTs greatly depend on the design effect, the sample size increase relative to an individually randomised trial. In diarrhoea CRTs, the design effect not only depends on the temporal and spatial variation of diarrhoea between clusters (which can be considerable [16]) but also on the number of follow-up surveys and the within-person correlation of diarrhoea, making the design effect difficult to predict [17]. We chose the proportion of days with diarrhoea (longitudinal prevalence) as the outcome for the sample size calculation [17]. Based on data from another ongoing study in Orissa (Boisson, unpublished), we assumed a mean longitudinal daily prevalence of 4% in children under 5, with a standard deviation of 7.6% assuming 6 follow up visits per child [18]. We assumed a 25% reduction in diarrhoea prevalence as a figure of public health interest and in line with estimates from systematic reviews [2,4-8]. Assuming 25 children per cluster, an intra-cluster correlation (ICC) of 0.025, a design effect of 1.6, and 10% loss to follow-up, 80% power and p=0.05 resulted in 50 clusters per arm. This figure was confirmed using a simulation method developed for the sample size estimation of complex trials [19].

### Eligibility criteria, enrolment and randomisation

Village-level eligibility criteria were: (i) sanitation coverage less than 10%; (ii) improved water supply; and (iii) no other water, sanitation or hygiene interventions anticipated in next 30 months. While we envisioned requiring that each eligible village have its own primary school, that requirement was dropped prior to village selection in order to meet the sample size. 28 out of 100 villages did not have a primary school at the time of enrolment. Households within villages were eligible if there was a child under 4 years (verified with immunization card), or a pregnant woman. Informed written consent was obtained from the male and/or female head of the household. We administered a questionnaire to each female head of enrolled household (demographics, socio-economic characteristics, water, hygiene and sanitation). Study villages were then randomly allocated to intervention and control stratified by block ensuring overall equal numbers of clusters in both arms. We follow an open cohort design. Households with a new baby born during the surveillance phase are also enrolled. Randomisation achieved a fair balance of socio-economic and water/sanitation related characteristics (Table 1).

**Table 1:** Socio-economic characteristics of study households at baseline survey (n= 1992)

**Characteristics**	**Intervention**	**Control**
Average persons per HH (SD)	6.4 (2.8)	6.3 (2.8)
Education level HH head, %		
None	27	31
Primary school not completed	22	19
Primary school completed	39	34
Some secondary school	12	17
Education level caregiver, %		
None	17	17
Primary school not completed	14	12
Primary school completed	50	50
Some secondary school	18	21
Caste, %		
Scheduled caste	18	22
Scheduled tribe	1	0
Other backward caste	39	35
Other caste	23	25
No information	18	18
Has BPL card	42	45
House structure		
Cement wall and roof (Pucca)	42	37
Cement wall (semi Pucca)	21	20
No cement (Kuchha)	37	43
Electricity, %	79	73
Owns agricultural land, %	76	74
Owns poultry/livestock, %	59	59
Water source, %		
Piped water	3	4
Deep tube well	38	39
Shallow tube well	41	44
Open well	9	2
River/lake/pond/canal	5	7
Other	4	4
Location of water source, %		
In own dwelling	18	15
In own compound	13	12
Outside compound	70	73
Access to a latrine, %	10	11

HH, household; BPL, below poverty line, certified by a government-issued card.

### Intervention

The intervention is a rural sanitation campaign conducted under the umbrella of the Government of India’s updated Total Sanitation Campaign (TSC) that follows a 'community led' and 'people centred' programme approach [13]. Emphasis is on awareness and demand generation for sanitation in households and schools, although in practice this is not always followed through [11]. Implementing agencies are bound by these guidelines, although there is flexibility to adapt TSC to local situations. WaterAid India (part of WaterAid, a major international NGO involved in water, sanitation and hygiene) and United Artists Association (an Orissa-based sanitation and water NGO) are coordinating TSC implementation in Puri. The intervention in Puri is funded by the Indian Government, European Union, and (for the trial only) SHARE, a UK government-funded research consortium on sanitation and hygiene.

Six local NGOs were contracted by the coordinating partners to deliver the intervention in collaboration with local governments (Gram Panchayat). The local NGOs are in charge of (1) community mobilisation through village motivators (1 for every 2 villages), (2) formation of Village Water and Sanitation Committees, youth and elders groups, (3) training of local masons, and (4) coordinating the supply chain for construction material. During the construction period, the government of India provides incentives (INR 2200 (US $44) to BPL households for latrines that meet specified criteria; subsequent revisions to the programme increased the subsidy and the specifications for qualifying latrines [13]. Water Aid India provides equivalent funding for certain priority households that are above the poverty line (APL) such as widow-headed households, and households with a disabled member. Other APL households are also encouraged to build latrines.

**Figure 3:**
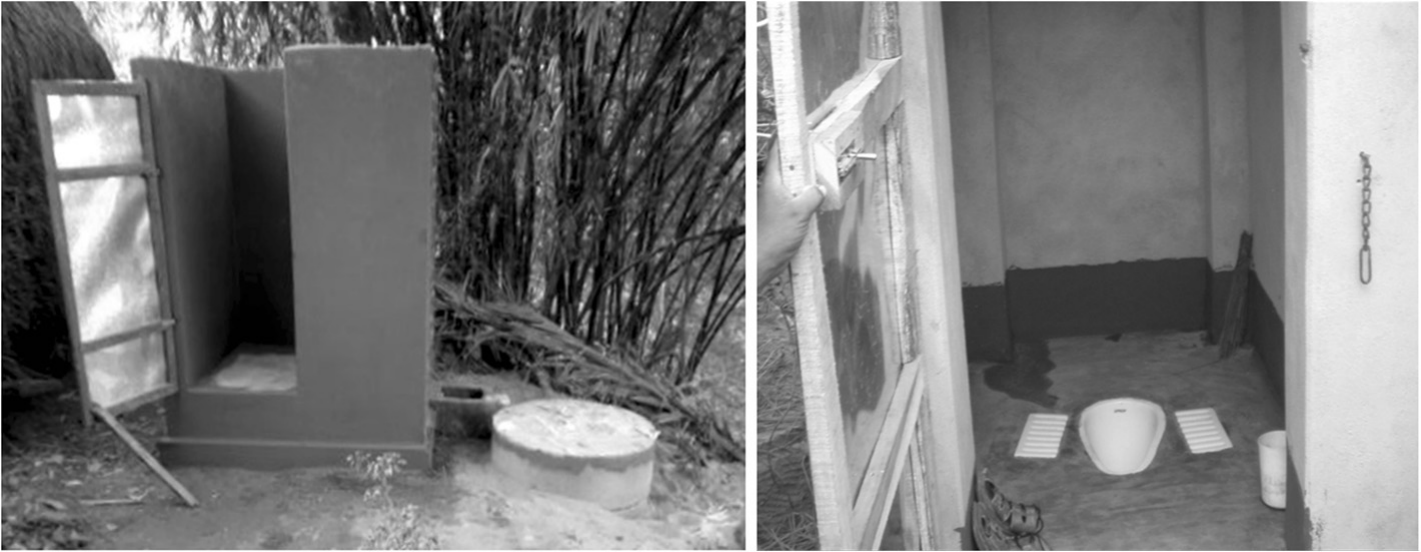
Photos of pour-flush latrines constructed in intervention villages (T. Clasen).

There is only one latrine option: a pour-flush (water seal) latrine with a single pit and Y-joint for a future second pit (Figure 3). Households are required to contribute sand, brick or stone and labour.

### Process documentation of intervention delivery

A process documentation team separate from the outcome assessment team and the implementing NGOs is visiting each intervention village every 4 to 8 weeks to document intervention delivery by conducting (i) direct observations of latrine status, (ii) interviews with the NGO-appointed Village Motivators and examination of local implementation documents, and (iii) interviews with members of the Village Water and Sanitation Committee. These data will be independently compared with the subsidy claim reports for latrine construction.

### Measuring uptake and compliance

We defined “sanitation coverage” as the proportion of households who have access to a latrine regardless of when it was built. This also includes shared latrines that householders report using. “Uptake” is the proportion of households that have a functional latrine at the end of the implementation period. Construction of latrines does not guarantee actual use (“compliance”) [11,20]. We assess compliance by recording reported latrine use, the condition and maintenance of the latrine, indicators of use (worn path to the facility, odour, flies, wet slab, cleaning materials) [21]. We also assess latrine use through “passive latrine use monitors (PLUMs)”—battery-powered devices that use door switches and infrared motion detector technology designed to objectively detect and record latrine visits for a period of 14 days per household [22]. Other methods complementing these data include in-depth interviews, focus group discussions and questionnaire surveys.

### Health outcome assessment

#### Reported diarrhea

Reported diarrhoea is a subjective outcome. It has been shown that frequent contacts with participants can lead to reporting fatigue leading to a general decline in prevalence over a study [23], and possibly bias [24]. We restricted the number of diarrhoea follow up visits to nine. Because delays in the latrine construction did not result in reaching the target coverage until January 2012 data from the first two diarrhoea surveys conducted between September and December 2011 will not be included in the primary analysis. We obtained an extension of our research grant that will allow follow up to continue until October 2013.

Originally, we chose daily point prevalence over the past three days as the main outcome. However, data from an ongoing study in the area (S. Boisson, unpublished) suggested that diarrhoea in children under 5 may be lower than expected. Unable to increase the sample size any further, we switched to seven-day period prevalence as the primary outcome measure to compensate for the potential loss in study power. Seven day period prevalence is a suitable outcome for interventions expected to primarily reduce incidence rather than disease duration [17], providing more statistical power than point prevalence data [18]. We defined diarrhoea according to WHO (three or more loose stools in 24 hrs [25]), a definition that may be the best compromise between external and internal validity [17]. The diarrhoea questions underwent extensive pilot testing based on local diarrhoea terms. Households are not asked to keep a diary of diarrhoea since the motivation to update diaries varies greatly. However, the fieldworkers use a visual aid showing a simple 10-day calendar to help participants remember the timing of episodes. This approach appeared to reduce reporting errors.

#### Soil-transmitted helminth infection

Initially, we planned to ask all persons aged 5 to 30 years of enrolled households to supply a baseline and follow-up stool sample, but this was extended to ages up to 40 years because of a low response rate. The delayed intervention roll-out made the timing of the worm survey difficult. Because of logistics and compliance with worm medications, it was not possible to have a long time gap between the worm survey and deworming. If we had conducted the survey too early, there would have been ample opportunity for re-infection during the time when most of the intervention arm population are still exposed to unimproved sanitation. If conducted too late, some of the difference between intervention and control might have been reduced because the intervention was already in place for several months. We chose, pragmatically, to conduct stool sample collection and treatment in the middle of the construction period.

Our study staff categorically refused to collect stool samples. For the collection, we employed four non-Hindus. A problem specific to sanitation interventions could be that the availability of a latrine may influence the willingness of participants to give a stool sample. Pilot testing suggested that people going for open defection may be reluctant to be seen carrying a stool sample back to the house. However, the proportion of samples collected was similar in intervention and control (44% vs. 43%), as was the baseline total worm prevalence (17.6% vs. 17.0%), indicating no evidence of bias.

After baseline stool collection, field staff offered a single dose of Albendazole, a broad-spectrum anthelminthic to all members of participating households (other than pregnant women in the first trimester). The decision to take a baseline sample was made largely on statistical grounds. Worm infections commonly show a high degree of spatial clustering [26] (our baseline results confirmed this). Adjusting for worm prevalence at baseline may increase the power of the analysis of the intervention effect.

Studies have shown that at high average infection intensity egg counts, prevalence of intestinal helminth infection is insensitive to changes in transmission intensity [27]. Our baseline data showed that the combined prevalence of hookworm (typically *Ancylostoma duodenale* and *Necator americanus*), roundworm (*Ascaris lumbricoides*), and whipworm (*Trichuris trichiura*) may be between 10 and 20%, with low to moderate average egg counts. In this range of infection intensity, we assumed prevalence at follow-up to be a suitable outcome and specified it as the main helminth outcome with intensity of infection to be used for exploratory analyses. We had no strong assumption on whether sanitation would affect worm species differently, and therefore specified the combined prevalence of hookworm, ascaris and trichuris as the main outcome.

Laboratory staff processes the samples using the ethyl-acetate sedimentation method [28]. We also considered using the Kato-Katz method but due to the large area covered by the study, lacked the laboratory logistics to conduct diagnostic tests on the same day. The ethyl-acetate sedimentation technique is known to underestimate egg counts but is deemed acceptable for measuring prevalence.

#### Anthropometrics

We measure the height (length) of all children < 2 years in participating households before completion of the intervention and at the end of the study following standard procedures for anthropometric assessment [29]. All children < 5 years will be weighed at each diarrhoea surveillance visit. Height and weight will be converted into z-scores (HAZ, WAZ) [30]. We assume that only a strong reduction in the exposure to faecal pathogens will lead to a measurable impact of the intervention on HAZ. It is unclear whether the “real-life” intervention evaluated in this study will achieve this during the timeframe of the follow-up. HAZ is often regarded as the better nutrition marker than WAZ, because inappropriate nutrition may increase weight without making the child healthier. This is less of a concern in a sanitation intervention aiming at improving nutritional status by reducing gastrointestinal infections, because any weight gain due to fewer infections may be regarded as beneficial. We measure WAZ repeatedly in each child as an indicator of recent diarrhoea [31]. We conduct back-checks on weight measurements in approximately 5% of the households selected at random. The repeated measure was carried out within 1 hour of previous weight measurement. Height measurements are repeated in a sample of 50 children at baseline and follow up [32]. Weight measurements are conducted with scale SECA 385, with 20 g increment for weight below 20 Kg and increment of 50 g for weight between 20–50 Kg. Recumbent length was measured with SECA 417 boards with 1mm increments. In a small number of households, participants refused weight measurements because of the fear that a child may lose weight by placing it on a scale.

### School attendance

We collect data on primary school attendance to explore the effect of the intervention on absenteeism. We assess attendance based on school records (which may be unreliable in some schools), parent report during household visit and school roll calls on the date of our three-monthly surveillance visits to the villages [33]. School attendance in this setting is not a straightforward study outcome because only a small fraction of absence episodes (less than 15%) appears to be related to infections preventable by sanitation. Systematically ascertaining reasons for school absence is difficult and beyond the scope of our study.

### Cost and cost-effectiveness analysis

We assess costs of the intervention and potential cost-savings associated with the intervention based on full economic costing from a societal perspective. Cost and cost savings at the household level are collected via household surveys. Cost data at the implementer level are collected through interviews and examination of the financial records of the implementing organizations. We will use effectiveness data from the trial outcomes to estimate the cost-effectiveness of the intervention, expressed in cost per disability-adjusted life years (DALYs) averted.

### Intermediate outcomes: exposure to excreta

Transmission of faecal pathogens is thought to occur via drinking contaminated water, person-to-person contact (e.g. hands, fomites), contaminated food, mechanical vectors (e.g. flies) and contact with soil [34]. In order to assess the extent to which the intervention reduces exposure to excreta-related pathogens we will compare intervention and control villages with respect to suitable indicators of each of the different pathways, as described below.

#### Drinking water

At each 3-monthly surveillance visit, we collect water samples from sources (well, communal tap, etc.) identified by participating households in the village as a drinking water source. Research has found water stored in households to frequently be more highly contaminated with faecal indicator bacteria (FIB) than source water, although the relevance of recontamination for health is unclear [35]. We hypothesize that a general reduction in environmental exposure to faecal bacteria due to the intervention may also reduce recontamination of stored water. On each visit we collect household water samples from 20% of participating households (randomly selected). Samples are placed in a cooler for transport and processed within 4 hours of collection using the membrane filtration technique, all in accordance with Standard Methods [36]. Ten percent of samples are processed in duplicate and 1 blank control for each day of processing.

#### Sanitary surveys

Every six months of follow-up, enumerators conduct unannounced sanitary surveys of all villages using a uniform survey methodology. The survey records the presence of faeces within a perimeter of each home randomly selected for this purpose, and reported data on child faeces disposal. The survey will also include the systematic observation of the use of open defecation sites, using methods we previously piloted in villages not part of the study. To conduct the observation we locally recruit female residents who observe from a distance deemed non-intrusive the number of people using known open defection sites during a pre-specified time-window.

#### Microbial source tracking and environmental exposure to pathogens

Sanitation interventions may reduce exposure to human but not necessarily animal excreta. Animals are a known source of gastro-intestinal pathogens, although human-to-human transmission (directly or via environmental exposure) may dominate [37]. We apply microbial source tracking based on host-specific *Bacteroidales* assays to distinguish and quantify the extent of human versus animal sources of faecal contamination in water and on hands [38]. Samples are taken in a sub-sample of control and intervention villages to explore the impact of the intervention and conduct further exploratory analysis of the impact of sanitation coverage and compliance on exposure to human faeces in the public and domestic domains of transmission. Quantitative and non-quantitative polymerase chain reaction (PCR and qPCR) methods will be used to distinguish human from non-human fecal sources and to detect zoonotic and human pathogens attributable to poor sanitation such as cryptosporidium, *giardia lamblia*, rotavirus and campylobacter [39].

In order to further assess whether the intervention is associated with measurable changes in the exposure of young children, we will assess faecal indictor bacteria in a sub-sample of the study population using two methods. First, we will measure the contamination of hands using hand rinses or chromogenic agar plates that are inoculated by finger imprints from study participants. In addition, we will give sterile toys to families with small children to be recollected after a certain time and analyzed for faecal indicator bacteria.

#### Vectors

Flies such as *Musca domestica* and *Musca sorbens* can act as mechanical vectors of diarrhoea pathogens Fly control measures can protect against diarrhoea [40,41]. We explore whether improved sanitation impacts (i) the quantity of fly vectors in food preparation areas, and (ii) the extent to which flies that are present carry pathogens. Following pilot-testing, we deploy fly traps in intervention and control villages during dry and monsoon seasons for identification, incrimination and quantification of fly vectors. Pooled fly samples will be tested for *Escherichia coli, Vibrio cholerae, Shigella spp., Salmonella spp.,* and *Aeromonas spp* as bacteria potentially transmitted by flies. We further explore the impact of the intervention on mosquitoes that typically use latrines as breeding sites such as *Culex quinquefasciatus*, a known vector for lymphatic filariasis, which is a major health problem in Orissa. Female mosquitoes will be trapped in intervention and control villages using gravid traps [42] and tested for presence of *Wuchereria bancrofti* (the dominant filarial species in Orissa), using PCR after Dynalbead purification [43].

### Data analysis

The primary endpoint of the study (prevalence of 7-day period prevalence of diarrhoea in children under 5 years) and the secondary binary health outcomes (all age diarrhoea prevalence, helminths) will be done on an intention-to-treat basis, i.e. independent of actual sanitation uptake at village or enrolled household level. We will treat diarrhoea and helminth prevalence as a binary outcome using a log-binomial model (log link, binomial family) for the calculation of prevalence ratios [44]. Clustering at village level will be accounted for by generalised estimating equations. Continuous secondary health endpoints (HAZ, WAZ) will be analysed using mixed effects linear regression accounting for clustering at village level.

We will use geographic data to support a range of exploratory analyses accounting for actual latrine uptake, by georeferencing and mapping for each study household the number and proportion of households with functional latrines within different buffer zones to explore the relative effect of individual and neighbourhood level sanitation coverage.

## Discussion

This large rural sanitation trial provides a number of challenges in terms of intervention delivery, epidemiological methods, logistics and validity of the outcome measures. While much of the focus in rural sanitation is on increasing coverage in order to report progress on the MDG target, interventions will not reduce exposure or prevent disease unless they are adopted by the target population. As an evaluation of a sanitation intervention as actually delivered by a leading NGO working in accordance with a large-scale government programme, the study is designed to have a high level of external validity. However, the study is limited by the circumstances in which it is located and the manner in which the intervention is executed. Coordinating community mobilisation and construction of latrines as implemented by different local NGOs in 50 randomly selected villages proved a challenge, with coverage increasing at a lower rate than expected. In the intervention arm, we expect latrine coverage of about 60% during much of the follow up period. The community mobilisation was implemented by the different NGOs with varying intensity and success. However, based on previous reports from different states in India, it seems unlikely that TSC as implemented in the trial population differs greatly from what normally is achieved [11,20].

Roll-out over one or more years may appear as a long time for the purposes of an RCT, but is a short time for achieving sustained behaviour change and latrine use. Evaluating the true impact of sanitation at a point in time when use of latrines becomes socially accepted, or even a socially desirable behaviour in the target population may be beyond the scope of RCTs as a tool for health impact evaluation. Withholding sanitation from randomly allocated control villages for more than two years may be ethically and politically unfeasible. The implementation of the intervention in the control villages means losing them for direct between-arm comparison. In this situation, some causal inference may be achieved by interpreting the disease trends in both arms following the intervention [45,46], but the strength of the evidence will be weaker. Since TSC programme delivery after the study is outside our control, we do not know to what extent (if at all) control villages will receive the intervention eventually. Contingent on additional funding, we are considering continuing follow-up of diarrhoea and worm infection and exploring the effect of increasing latrine coverage in the control arm, using analytical approaches that exploit the differences in latrine uptake over time.

Socially sensitive interventions such as those targeting sanitation or hygiene may be particularly prone to reactivity in the study participants, especially when responding to survey questions. There is a potential for bias in subjective outcomes such as reported diarrhoea [24]. Throughout the trial we aim at minimising the extent to which the study population would be exposed to the trial procedures. Because the teams in charge for the different activities and outcomes are visiting the villages separately, most villagers do not make a direct link between the health outcome measurement and the latrine construction. This is aided by the fact that many NGOs are working in the area for different purposes. This risk of bias will be evaluated through qualitative and quantitative data towards the end of the study, including comparisons of subjective health outcomes (reported diarrhoea) with objective health outcomes (anthropometrics, helminth infection), with other non-health outcomes (school attendance) and with intermediate environmental outcomes that assess the impact of the intervention on exposure. Analysis of associations between self-reported outcomes and intervention coverage and uptake may also be helpful in exploring bias.

Four considerations may be especially important in designing evaluations of sanitation interventions in order to deal with the complexity of the subject. First, process evaluation - documenting the manner in which the intervention is actually implemented rather than intended or reported by the program implementers - is essential to put health outcomes into perspective. Similarly, measuring latrine use (compliance) is critical since lack of use even by a minority of the population may compromise the potential health impact. Third, because even effective sanitation may be insufficient in preventing diarrhoeal disease and helminth infection, it is necessary to assess whether the intervention has reduced exposure to pathogens, especially if no health impact is found. In our study we attempt to achieve this by looking at the main transmission pathways suggested by the so-called F-Diagram [34], such as fluids (water), fields (food and surrounding environment), flies (mechanical vectors) and fingers (hand hygiene). Fourth, unlike handwashing with soap or household water treatment where a household might protect itself by high compliance, sanitation may require compliance by one’s neighbours as well. We will conduct spatial analysis to explore the effect of density of uptake as well as any spillover impact on non-adopters.

## Conclusion

In conclusion, evaluating a sanitation campaign using an RCT design requires difficult decisions at various steps. However, the policy implications of the trial results (expected in late 2013) may need to be considered with similar caution. While a positive result may help to encourage policy makers to increase their commitment to rural sanitation, a negative result may achieve the opposite if the possible reasons for programme failure are not fully documented and taken into consideration.

## Competing interests

The authors declare that they have no competing interests.

## Authors’ contributions

All authors contributed to the conception of the study described in the paper. TC and WS drafted the original manuscript. All authors commented on successive drafts of the manuscripts and read and approved the final manuscript.
